# COVID-19 Risk Factors for Cancer Patients: A First Report with Comparator Data from COVID-19 Negative Cancer Patients

**DOI:** 10.3390/cancers13102479

**Published:** 2021-05-19

**Authors:** Beth Russell, Charlotte L. Moss, Kieran Palmer, Rushan Sylva, Andrea D’Souza, Harriet Wylie, Anna Haire, Fidelma Cahill, Renee Steel, Angela Hoyes, Isabelle Wilson, Alyson Macneil, Belul Shifa, Maria J Monroy-Iglesias, Sophie Papa, Sheeba Irshad, Paul Ross, James Spicer, Shahram Kordasti, Danielle Crawley, Kamarul Zaki, Ailsa Sita-Lumsden, Debra Josephs, Deborah Enting, Angela Swampillai, Elinor Sawyer, Paul Fields, David Wrench, Anne Rigg, Richard Sullivan, Mieke Van Hemelrijck, Saoirse Dolly

**Affiliations:** 1 Translational Oncology and Urology Research (TOUR), School of Cancer and Pharmaceutical Sciences, King’s College London, London SE1 9RT, UK; charlotte.moss@kcl.ac.uk (C.L.M.); harriet.wylie@kcl.ac.uk (H.W.); anna.haire@kcl.ac.uk (A.H.); Fidelma.Cahill@gstt.nhs.uk (F.C.); maria.j.monroy_iglesias@kcl.ac.uk (M.J.M.-I.); danielle.crawley@kcl.ac.uk (D.C.); Debra.Josephs@gstt.nhs.uk (D.J.); deborah.enting@kcl.ac.uk (D.E.); 2 King’s College Hospital NHS Foundation Trust, London SE5 9RS, UK; kieran.palmer3@nhs.net; 3 Medical Oncology, Guy’s and St Thomas’ NHS Foundation Trust (GSTT), London SE1 9RT, UK; Rushan.Sylva@gstt.nhs.uk (R.S.); Andrea.DSouza@gstt.nhs.uk (A.D.); sophie.papa@kcl.ac.uk (S.P.); sheeba.irshad@kcl.ac.uk (S.I.); Paul.Ross@gstt.nhs.uk (P.R.); james.spicer@kcl.ac.uk (J.S.); Kamarul.Zaki@gstt.nhs.uk (K.Z.); Ailsa.Lumsden@gstt.nhs.uk (A.S.-L.); Anne.Rigg@gstt.nhs.uk (A.R.); saoirse.dolly@gstt.nhs.uk (S.D.); 4 Clinical Oncology, Guy’s and St Thomas’ NHS Foundation Trust (GSTT), London SE1 9RT, UK; Renee.Steel@gstt.nhs.uk (R.S.); Angela.Swampillai@gstt.nhs.uk (A.S.); elinor.sawyer@kcl.ac.uk (E.S.); 5 Haematology Department, Guy’s and St Thomas’ NHS Foundation Trust (GSTT), London SE1 9RT, UK; Angela.Hoyes@gstt.nhs.uk (A.H.); shahram.kordasti@kcl.ac.uk (S.K.); Paul.Fields@gstt.nhs.uk (P.F.); David.Wrench@gstt.nhs.uk (D.W.); 6 Guy’s and St Thomas’ NHS Foundation Trust (GSTT), London SE1 9RT, UK; Isabelle.Wilson@gstt.nhs.uk; 7 Breast Surgery, Guy’s and St Thomas’ NHS Foundation Trust (GSTT), London SE1 9RT, UK; alymacneil@gmail.com (A.M.); Belul.Shifa@gstt.nhs.uk (B.S.); 8 School of Cancer and Pharmaceutical Sciences, King’s College London, London SE1 9RT, UK; richard.sullivan@kcl.ac.uk

**Keywords:** COVID-19, cancer, risk factors

## Abstract

**Simple Summary:**

The COVID-19 pandemic has had a detrimental impact on cancer patients globally. Whilst there are several studies looking at the potential risk factors for COVID-19 disease and related death, most of these include non-cancerous patients as the COVID-19 negative comparator group, meaning it is difficult to draw hard conclusions as to the implications for cancer patients. In our study, we utilized data from over 2000 cancer patients from a large tertiary Cancer Centre in London. In summary, our study found that patients who are male, of Black or Asian ethnicity, or with a hematological malignancy are at an increased risk of COVID-19. The use of cancer patients as the COVID-19 negative comparator group is a major advantage to the study as it means we can better understand the true impact of COVID-19 on cancer patients and identify which factors pose the biggest risk to their likelihood of infection with SARS-CoV2.

**Abstract:**

Very few studies investigating COVID-19 in cancer patients have included cancer patients as controls. We aimed to identify factors associated with the risk of testing positive for SARS CoV2 infection in a cohort of cancer patients. We analyzed data from all cancer patients swabbed for COVID-19 between 1^st^ March and 31^st^ July 2020 at Guy’s Cancer Centre. We conducted logistic regression analyses to identify which factors were associated with a positive COVID-19 test. Results: Of the 2152 patients tested for COVID-19, 190 (9%) tested positive. Male sex, black ethnicity, and hematological cancer type were positively associated with risk of COVID-19 (OR = 1.85, 95%CI:1.37–2.51; OR = 1.93, 95%CI:1.31–2.84; OR = 2.29, 95%CI:1.45–3.62, respectively) as compared to females, white ethnicity, or solid cancer type, respectively. Male, Asian ethnicity, and hematological cancer type were associated with an increased risk of severe COVID-19 (OR = 3.12, 95%CI:1.58–6.14; OR = 2.97, 95%CI:1.00–8.93; OR = 2.43, 95%CI:1.00–5.90, respectively). This study is one of the first to compare the risk of COVID-19 incidence and severity in cancer patients when including cancer patients as controls. Results from this study have echoed those of previous reports, that patients who are male, of black or Asian ethnicity, or with a hematological malignancy are at an increased risk of COVID-19.

## 1. Introduction

Whilst the COVID-19 research and innovation landscape has led to a plethora of publications in the context of cancer, we still do not have a good understanding of what may make some cancer patients more likely to get infected with SARS-CoV-2. To our knowledge, most studies to date discuss the risk factors for COVID-19 in the general population, as described recently in a meta-analysis by Pijls et al. [1]. Based on 59 studies, including 36,470 patients, male sex and age >70 were found to be consistently associated with a higher risk of COVID-19, severe disease, intensive care unit (ICU) admission, and death [1].

In the context of cancer, several cohort studies presented the clinical and demographic characteristics of cancer patients diagnosed with SARS-CoV-2 infection and/or their association with COVID-19 outcomes [2,3]. However, the true rate of COVID-19 disease in oncology patients remains unquantified because the denominator is not known, i.e., the actual number of all cancer patients infected with SARS CoV-2 [4]. We have previously reported on the scale of COVID-19 infection in cancer patients based on 1 week of COVID-19 testing in our Cancer Centre and identified that 1.38% of cancer patients tested positive for COVID-19 [5]. We have now completed this data, which provides us with the unique opportunity to identify which factors are associated with an increased risk of SARS-CoV-2 infection in cancer patients, a question which hitherto has not been investigated due to the lack of data on a comparator, i.e., cancer patients who tested negative for SARS-CoV-2 infection.

In this study, we aimed to describe factors associated with the risk of COVID-19 in cancer patients, whilst using cancer patients with a COVID-19 negative test as the comparator.

## 2. Materials and Methods

Our Centre in South-East London, treating approximately 8800 patients annually (including 4500 new diagnoses), is one of the largest comprehensive cancer Centers in the UK and was at the epicenter of the UK COVID-19 epidemic during the first wave. We reported our first COVID-19 positive cancer patient on 29th February 2020. Until 30th April 2020, a COVID-19 swab was ordered for cancer patients with symptoms necessitating hospitalization or for those scheduled to undergo a cancer-related treatment. From 1st May 2020 until mid-June 2020, COVID-19 testing was introduced as a standard of care, with about 25% of patients being swabbed daily depending upon staff and testing kit availability [5]. COVID-19 was categorized based on the World Health Organization (WHO) criteria for disease severity [6], and we included those who died from COVID-19 in the severe group. A detailed analysis of the COVID-19 positive cancer patients (29th February until 30th June 2020) at our center was published elsewhere and focuses specifically on the cancer patient characteristics indicative of COVID-19 severity and death [7,8].

Here, we analyzed data from 1st March to 30th June 2020 for COVID-19 test results in all cancer patients at our center. All data was collected and analyzed as part of Guy’s Cancer Cohort (Ethics Reference number: 18/NW/0297) [9], a research ethics committee-approved research database of all routinely collected clinical data of cancer patients diagnosed or treated at Guy’s and St Thomas’ (GSTT) NHS Foundation Trust. Over 83% of patients filled out a symptom assessment form, of which 82% were asymptomatic. Based on their demographics and tumor characteristics, this sample can be considered to be representative of the total population.

### Statistical Analyses

Descriptive statistics were used to describe the demographic and clinical characteristics of the patients based on COVID-19 status. Socio-economic status (low, middle, high) was categorized based on the English Indices of Multiple Deprivation for postcodes [10]. Radical treatment referred to those patients with a chance of long-term survival or cure.

**Figure A1:**
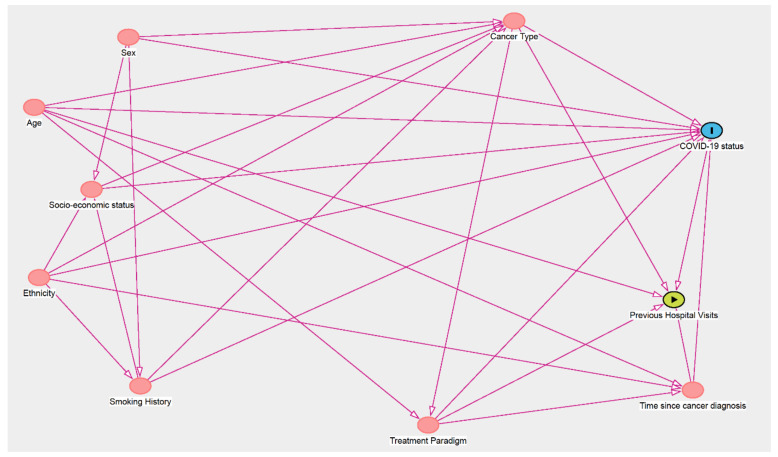
Directed Acyclic Graph (DAG) for the association between demographic and clinical factors and risk of COVID-19 in cancer patients (www.dagitty.net, accessed on 11 January 2021).

We conducted logistic regression analyses to identify which factors were associated with a positive COVID-19 test. Additional analyses were conducted, whereby a positive COVID-19 test was further categorized into mild/moderate or severe disease. Pneumonia with or without sepsis (i.e., those patients managed on the ward) was an indicator of mild/moderate COVID-19, whereas acute respiratory distress syndrome (ARDS), septic shock (i.e., those patients whose severity reached criteria for Intensive Care Unit admission, if deemed clinically appropriate), or COVID-related death were indicators of severe COVID-19, as defined by the WHO COVID-19 classification [6]. We used a directed acyclic graph (DAG) (Figure A1 in Appendix A) to inform the models to quantify the association between each factor and COVID-19 status. Each factor was individually set as the main exposure variable in the model when determining the minimal adjustments required (Table A1 in Appendix A).

## 3. Results

### 3.1. Cohort Demographics

Of the 2152 patients included in the study, 190 patients (9%) tested positive for COVID-19 (Table 1), of which 34 (18%) were asymptomatic. Overall, there were slightly more females than males in the cohort (55% vs 45%, respectively); however, in the COVID-19 positive cohort, there was a higher proportion of males compared to females (59% vs 41%, respectively). The age groups were fairly similarly distributed between the COVID-19 positive and negative patients. The mean age in the COVID-19 positives was 63.80 (SD 14.80), whilst the mean age in the COVID-19 negatives was 62.50 (SD 13.20). The majority of patients were of a low SES (86%). Overall, 12% of patients were black and just under 3% were Asian. When stratified by COVID-19 status, 22% were black in the COVID-19 positive group compared to under 12% in the negative patients.

In terms of cancer characteristics, the most common tumor type was breast in both the overall (21%) and COVID-19 negative patients (22%). However, in the COVID-19 positive group, the largest proportion of patients had a urological tumor (22%). Hematological cancers were the second most common in the COVID-19 positive group, present in 17% of patients. With respect to the treatment paradigm, in the overall cohort, palliative treatment was the most common (45%), followed by adjuvant (20%) and radical treatment (18%). There was a higher proportion of patients who were treatment-naïve in the COVID-19 positive group compared to the COVID-19 negative patients (10% vs 2%, respectively). Of the patients on palliative treatment, the majority of patients were either on first- or second-line treatment. For those patients on systemic anticancer therapy (SACT), the most common type was systemic chemotherapy (45%); this remained true when stratified by COVID-19 status. Whilst the highest proportion of patients had their cancer diagnosed just under a year prior to their test for COVID-19, the median time from cancer diagnosis was 13 months (IQR:4, 37 months). However, for those with COVID-19, , the median time since cancer diagnosis was 9 months (IQR:2–45 months) compared to 14 months (IQR:4, 37 months) in those patients who did not have COVID-19.

### 3.2. Risk of Developing COVID-19

Males were at an increased risk of being diagnosed with COVID-19 compared to females (OR = 1.85, 95%CI:1.37–2.51) (Table 2). Patients of black ethnicity (OR = 1.93, 95%CI:1.31–2.84) and those with a hematological cancer type (OR = 2.29, 95%CI:1.45–3.62) were at an increased risk of having a positive COVID-19 result compared to those of white ethnicity and those with solid malignancies, respectively. Patients who were on either radical or palliative treatment appeared to be at a lower risk of COVID-19 compared to patients on no active treatment (Radical, OR = 0.37, 95%CI:0.20–0.66 and palliative, OR = 0.39, 95%CI:0.22–0.70).

### 3.3. Risk of COVID-19 Severity

Males were at an increased risk of both mild/moderate and severe COVID-19 when compared to females with OR = 1.62 (95%CI:1.16–2.27) for mild/moderate and OR = 3.12 (95%CI:1.58–6.14) for severe disease (Table 3). For ethnicity, black patients were at an increased risk of mild/moderate disease when compared to white patients (OR = 2.16, 95%CI:1.41–3.31). However, this association did not remain a risk of severe COVID-19 (OR = 1.26, 95%CI:0.54–2.95). Patients of Asian ethnicity had, at a borderline, significantly increased risk of severe COVID-19 only (OR = 2.97, 95%CI:1.00–8.93).

When compared to solid malignancies, those with hematological malignancies were at a significantly increased risk of mild/moderate (OR = 2.30, 95%CI:1.38–3.83) and severe COVID-19 (OR = 2.43, 95%CI:1.00–5.90), though the latter was borderline significant. Patients undergoing radical/curative and palliative treatment were at a decreased risk of mild/moderate COVID-19 when compared to patients on no active treatment (Radical/curative, OR = 0.37, 95%CI:0.20–0.69; Palliative, OR = 0.36, 95%CI:0.19–0.66). However, they did not, appear to have a significantly different risk of severe COVID-19. Patients who had been diagnosed with their cancer over 5 years before were at an increased risk of severe COVID-19 when compared to those who had been diagnosed within 1 year (OR = 2.89, 95%CI:1.12–7.50).

## 4. Discussion

To the best of our knowledge, this is one of the first studies to assess the risk of COVID-19 in a real-world cancer patient population, using the COVID-19 negative group as controls for those with COVID-19. Our results show that cancer patients who are male, of black ethnicity and those with a hematological cancer type were at a significantly increased risk of COVID-19 and more specifically mild/moderate disease. Furthermore, cancer patients who are male, of Asian ethnicity, with a hematological cancer type and those diagnosed with cancer over 5 years ago were at a significantly increased risk of severe COVID-19. The results from this study corroborate with those of our previously reported results when looking at the risk of COVID-19 severity and death in a cohort of COVID-19 positive cancer patients [7,8].

We previously reported a prevalence of 1.4% of COVID-19 in our cancer population, a figure taken from 1 weeks’ worth of testing as standard care at our center [5]. In the current study, 9% of our cancer population tested positive for COVID-19 over the 5 months of this study. This figure is a reflection of the targeted testing carried out at our center. However, it is also possible that, during the latter months, some patients were being tested and diagnosed with COVID-19 at their local testing centers; therefore, we may not have captured all COVID-19 positive cancer patients under our care. A previous study, which looked at the prevalence of COVID-19 in a population of cancer patients, found that 18% of patients who had suspected COVID-19 had detectable SARS-COV2 infection [11]. This higher proportion of detected cases compared to our study may be somewhat explained by the reasons for testing. In the study by Assaad et al., patients were only tested if COVID-19 was suspected. However, at our center, patients underwent testing for COVID-19 infection for numerous reasons over the study period, including screening for treatment, such as having surgery, being symptomatic, and due to routine testing. Only 17% of our patients were symptomatic or deemed clinically or radiologically suspicious for COVID-19. It is also worth noting that our data was collected at the height of the first wave of the COVID-19 pandemic when the UK was in national lockdown and, therefore, most of our patients were shielding. These factors may have influenced our lower infection rate compared to that of Assaad et al. in France [11].

Males have consistently been observed to be at an increased risk of COVID-19. In their systematic review, comprising 17 studies, Park et al. reported an OR of 1.60 (95%CI:1.38–1.85) for the composite of severe COVID-19 and all-cause death for males compared to females [12,13]. The current study has shown that this increased risk for males also stands true for patients with cancer. This is an important finding as most studies do not look at cancer patients specifically. Biological mechanisms surrounding the role of ACE2 and TMPRSS2 in males enhancing the viral entry and invasion of cells have been proposed, with increasing evidence to support this [12,14].

Here, we also report that cancer patients of black ethnicity were at an increased risk of developing COVID-19, albeit mild/moderate infection, compared to patients of white ethnicity. We also report an increased risk of severe COVID-19 for patients of Asian ethnicity compared to those of white ethnicity. These results concur with our preceding report on COVID-19 positive patients only [7,8]. In this previous study, we found that Asian patients, but not black patients, were at an increased risk of severe COVID-19 (compared to mild/moderate disease) when compared to white patients. Black, Asian, and minority ethnic (BAME) individuals have repeatedly been over-represented within non-cancerous COVID-19 cohorts [15,16,17,18,19]. One study, performed using data from the UK biobank, investigated whether factors, such as deprivation, cardiometabolic morbidities, and 25(OH)-vitamin D levels, attenuated the association of ethnicity with COVID-19 status [15]. Similar to our study, they found no significant association with deprivation and risk of COVID-19 and further concluded that these factors did not explain the strong association with ethnicity [15].

Several published studies, including our own, have reported worse outcomes and higher mortality rates in patients with hematological malignancies compared to solid cancers [7,20,21,22]. Whilst we did not look at mortality in this current study, we found that patients with a hematological malignancy were at a two-fold increased risk of severe COVID-19, thus complimenting the results from other studies. A recent, yet to be published, study delved into potential reasons behind this association [22]. The researchers found that patients with hematological malignancies had an impaired SARS-CoV2-specific antibody response when compared to those with solid malignancies; an observation also noted by Abdul-Jawad et al. [22,23]. They further concluded that, in the absence of a humoral response, CD8 T cells were critical for the survival of hematological cancer patients with COVID-19 [22]. The authors explain that this immune response may impact the response that hematological patients have to the COVID-19 vaccines, thus highlighting this as a vital area for future research.

Being on a curative or palliative treatment paradigm was found to be associated with a decreased risk of COVID-19. This interesting result may in part be explained by the behavior of cancer patients. Many cancer patients were either asked to or chose to shield when the UK went into lockdown in March 2020. As a result, patients undergoing active treatment, whether this be curative or palliative, may have been protected from COVID-19 due to a reduced exposure. On the contrary, the study by Assaad et al. found a higher proportion of patients undergoing cancer treatment in the past month in the COVID-19 positive patients compared to those who remained COVID-19 free (*p* = 0.049) [11]. Lee et al. reported that cancer patients who had undergone chemotherapy in the 4 weeks prior to testing positive for COVID were not at risk of increased mortality from COVID-19 [24]. In our previous study, we also reported that patients on palliative treatment were at increased risk of being diagnosed with severe COVID-19 (compared to mild/moderate disease) and COVID-19-related death [7,8].

A strength of this study is the use of COVID-19-negative cancer patients as controls in our cohort. Previous studies have frequently used non-cancerous patients as controls [20,25,26,27,28] or performed case-control studies using non-cancerous patients only [1,15]. By using cancer patients as the control, we can better understand the true impact of COVID-19 on cancer patients and which factors pose the biggest risk to their likelihood of infection with SARS-CoV2. By including our entire cancer population, this can potentially minimize selection biases in comparison to reports including only the COVID-19 positive patients. Many of these studies include large multi-center consortiums, where the denominator of patients and their outcomes are unknown.

A limitation to the current study is the use of single center data. Having said this, we were still able to use data on a large population of patients (*n* = 2152). Moreover, as previously discussed, some COVID-19 positive cases may have been missed due to testing in the community or at external centers. We minimized the impact of this by cross-checking our data using Network Hospitals and Cancer Alliance networks. Despite rigorous internal validation of the dataset, a further limitation to the study is the proportion of missing data for certain variables, such as ethnicity (30%) and smoking status (44%).

In light of the results published by Monin-Aldama et al. [29], whereby the immune efficacy of the COVID-19 vaccine was increased with a booster vaccine within 21 days in cancer patients, future research (already underway) includes looking into the effects of the COVID-19 vaccination program on these cancer patients.

## 5. Conclusions

Data from this study, comparing both COVID-19 negative and positive cancer patients, has provided us with the unique opportunity to identify which factors are associated with an increased risk of SARS-CoV-2 infection in cancer patients. Results from this study have echoed those of previous reports that both demographic (sex and ethnicity) and clinical characteristics (type of malignancy) are associated with an increased risk of COVID-19. To the best of our knowledge, this is one of the first studies to utilize cancer patients as the comparator group for COVID-19 risk factors; hence, studies to date need to be considered carefully, since they often include non-cancer patients as the denominator. These results, together with data from our previous studies on mortality, can further help clinicians to identify their patients most at risk of COVID-19, thus giving them the opportunity to take appropriate actions to alleviate this risk. Future studies will also have to take into account the effects of COVID-19 vaccination programs, which are currently being rolled out across the globe [30].

## Figures and Tables

**Table 1:** Demographic and cancer characteristics of COVID-19 positive and negative cancer patients.

Variable	Total (*n* = 2152)		COVID-19 Status			
			Positive (*n* = 190)		Negative (*n* = 1962)	
	*n*	%	*n*	%	*n*	%
**Sex**						
Male	969	45.00	112	58.90	857	43.70
Female	1183	55.00	78	41.10	1105	56.30
**Age**						
<50	362	16.80	30	15.80	332	16.90
50–59	503	23.40	36	18.90	467	23.80
60–69	613	28.50	55	28.90	558	28.40
70–79	476	22.10	40	21.10	436	22.20
≥80	198	9.20	29	15.30	169	8.60
Mean (SD)	62.60 (13.30)		63.80 (14.80)		62.50 (13.20)	
**SES**						
Low	1848	85.90	157	82.60	1691	86.20
Medium	36	1.70	0	0.00	36	1.80
High	150	7.00	19	10.00	131	6.70
Missing	118	5.50	14	7.40	104	5.30
**Ethnicity**						
White British	923	42.90	84	44.20	839	42.80
White Other	196	9.10	14	7.40	182	9.30
Black Caribbean	89	4.10	10	5.30	79	4.00
Black African	102	4.70	17	8.90	85	4.30
Black Other	78	3.60	15	7.90	63	3.20
Asian	62	2.90	7	3.70	55	2.80
Mixed	25	1.20	2	1.10	23	1.20
Other	43	2.00	4	2.10	39	2.00
Unknown	634	29.50	37	19.50	597	30.40
**Smoking history**						
Never	563	26.20	66	34.70	497	25.30
Current	206	9.60	19	10.00	187	9.50
Ex-smoker	432	20.10	48	25.30	384	19.60
Unknown	951	44.20	57	30.00	894	45.60
**Cancer type**						
Urological	320	14.90	41	21.50	279	14.20
Gynaecological	226	10.50	10	5.30	216	11.00
Gastro-intestinal	395	18.40	29	15.30	366	18.70
Skin/Head & neck	199	9.20	16	8.40	183	9.30
Central Nervous System	39	1.80	12	6.30	27	1.40
Breast	456	21.20	27	14.20	429	21.90
Lung	300	13.90	22	11.60	278	14.20
Haematological	195	9.10	33	17.40	162	8.30
Other	22	1.00	0	0.00	22	1.10
**Cancer stage**						
I	233	10.80	21	11.10	212	10.80
II	304	14.10	34	17.90	270	13.80
III	450	20.90	34	17.90	416	21.20
IV	889	41.30	76	40.00	813	41.40
Missing	276	12.80	25	13.20	251	12.80
**Treatment Paradigm**						
Treatment naive	56	2.60	18	9.50	38	1.90
Neoadjuvant	117	5.40	10	5.30	107	5.50
Adjuvant	419	19.50	12	6.30	407	20.70
Radical	388	18.00	49	25.80	339	17.30
Palliative	970	45.10	78	41.10	892	45.50
Watch and wait	96	4.50	18	9.50	78	4.00
Missing	106	4.90	5	2.60	101	5.10
**Line of Palliative Treatment (*n* = 970)**						
1	370	38.60	38	50.70	332	37.60
2	243	25.40	22	29.30	221	25.00
3	108	11.30	7	9.30	101	11.40
>4	53	5.60	1	1.30	52	5.90
Missing	196	20.20	10	12.80	186	20.90
**Systemic Treatment (*n* = 1448)**						
Chemotherapy	708	48.90	65	61.90	643	47.90
Immunotherapy	110	7.00	9	8.60	101	7.50
Biological	163	11.30	14	13.30	149	11.10
Targeted Therapy	202	14.00	6	5.70	196	14.60
Combination Therapy	265	18.30	11	10.50	254	18.90
**Time since cancer diagnosis**						
<1 year	974	45.30	96	50.50	878	44.80
1–2 years	340	15.80	23	12.10	317	16.20
2–5 years	399	18.50	29	15.30	370	18.90
≥5 years	323	15.00	36	18.90	287	14.60
Missing	116	5.40	6	3.20	110	5.60

**Table 2:** Odds ratios and 95% confidence intervals for risk of COVID-19 in cancer patients.

Variable	COVID-19 Positive *n* (%)	COVID-19 Negative *n* (%)	OR *	95% CI
**Sex**				
Female	78 (41)	1105 (56)	1.00	Ref.
Male	112 (59)	857 (44)	1.85	(1.37–2.51)
**Age**				
≤60	66 (35)	799 (41)	1.00	Ref.
>60	124 (65)	1163 (59)	1.29	(0.94–1.76)
**SES**				
Low	157 (83)	1691 (86)	1.00	Ref.
Middle/High	19 (0)	167 (9)	1.58	(0.94–2.66)
**Ethnicity**				
White	98 (52)	1021 (52)	1.00	Ref.
Black	42 (22)	227 (12)	1.93	(1.31–2.84)
Asian	7 (4)	55 (3)	1.33	(0.59–2.99)
Other	6 (3)	62 (3)	1.01	(0.43–2.39)
**Smoking History**				
Never	66 (35)	497 (25)	1.00	Ref.
Ever	67 (35)	571 (29)	0.91	(0.61–1.37)
Cancer Type				
Solid	157 (83)	1778 (91)	1.00	Ref.
Haematological	33 (17)	162 (8)	2.29	(1.45–3.62)
**Tumour Stage**				
I-III	43 (23)	1103 (56)	1.00	Ref.
IV	17 (9)	302 (15)	1.44	(0.81–2.57)
**Treatment Paradigm**				
No active treatment	18 (0)	78 (4)	1.00	Ref.
Radical/Curative	71 (37)	853 (43)	0.37	(0.20–0.66)
Palliative	78 (41)	892 (45)	0.39	(0.22–0.70)
**Time since cancer diagnosis**				
<1 year	96 (51)	878 (45)	1.00	Ref.
1–2 years	23 (12)	317 (16)	0.70	(0.42–1.16)
2–5 years	29 (15)	370 (19)	0.73	(0.45–1.18)
≥5 years	36 (19)	287 (15)	1.11	(0.70–1.76)

* Adjusted models according to DAG in Appendix A.

**Table 3:** Odds ratios and 95% confidence intervals for risk of mild/moderate and severe COVID-19 in cancer patients.

Variable	COVID-19 Negative	Mild/Moderate COVID-19	Risk of Mild/Moderate		Severe COVID-19	Risk of Severe COVID-19	
	*n* (%)	*n* (%)	OR *	95% CI	*n* (%)	OR *	95% CI
**Sex**							
Female	1105 (56)	66 (44)	1.00	Ref.	12 (29)	1.00	Ref.
Male	857 (44)	83 (56)	1.62	(1.16–2.27)	29 (71)	3.12	(1.58–6.14)
**Age**							
≤60	799 (41)	54 (36)	1.00	Ref.	12 (29)	1.00	Ref.
>60	1163 (59)	95 (64)	1.21	(0.86–1.71)	29 (71)	1.66	(0.84–3.27)
**SES**							
Low	1691 (86)	124 (83)	1.00	Ref.	33 (80)	1.00	Ref.
Middle/High	167 (9)	16 (11)	1.64	(0.93–2.88)	3 (7)	1.38	(0.40–4.76)
**Ethnicity**							
White	1021 (52)	73 (49)	1.00	Ref.	25 (61)	1.00	Ref.
Black	227 (12)	35 (23)	2.16	(1.41–3.31)	7 (17)	1.26	(0.54–2.95)
Asian	55 (3)	3 (2)	0.76	(0.23–2.50)	4 (10)	2.97	(1.00–8.83)
Other	62 (3)	4 (3)	0.90	(0.32–2.55)	2 (5)	1.32	(0.31–5.69)
**Smoking History**							
Never	497 (25)	53 (36)	1.00	Ref.	13 (32)	1.00	Ref.
Ever	571 (29)	51 (34)	0.91	(0.58–1.42)	16 (39)	0.90	(0.38–2.10)
**Cancer Type**							
Solid	1778 (91)	126 (85)	1.00	Ref.	31 (76)	1.00	Ref.
Haematological	162 (8)	23 (15)	2.30	(1.38–3.83)	10 (24)	2.43	(1.00–5.90)
**Tumour Stage**							
I-III	1103 (56)	37 (25)	1.00	Ref.	6 (15)	1.00	Ref.
IV	302 (15)	15 (10)	1.48	(0.80–2.73)	2 (5)	1.22	(0.24–6.06)
**Treatment Paradigm**							
No active treatment	78 (4)	15 (10)	1.00	Ref.	3 (7)	1.00	Ref.
Radical/Curative	853 (43)	58 (39)	0.37	(0.20–0.69)	13 (32)	0.37	(0.10–1.38)
Palliative	892 (45)	59 (40)	0.36	(0.19–0.66)	19 (46)	0.56	(0.16–1.95)
**Time since cancer diagnosis**							
<1 year	878 (45)	83 (56)	1.00	Ref.	13 (32)	1.00	Ref.
1–2 years	317 (16)	18 (12)	0.63	(0.36–1.11)	5 (12)	1.27	(0.41–3.90)
2–5 years	370 (19)	20 (13)	0.59	(0.34–1.02)	9 (22)	1.69	(0.62–4.59)
≥5 years	287 (15)	24 (16)	0.87	(0.51–1.46)	12 (29)	2.89	(1.12–7.50)

* Adjusted models according to DAG in the Appendix A.

## Data Availability

The data presented in this study are available on request from the corresponding author. The data are not publicly available due to ethical reasons.

## Appendix A

**Table A1:** Overview of minimal adjustments for the associations between demographic and clinical characteristics and risk of COVID-19 or death in cancer patients (www.dagitty.net, accessed on 11 January 2021).

Main Exposure	Minimal Sufficient Adjustment sets for Estimating the Total Effect of the Exposure Variable on COVID-19 Status/Severity
Age	None
Sex	None
Socio-Economic Status	Ethnicity, Smoking History
Ethnicity	None
Smoking History	Ethnicity, Sex
Cancer Type	Age, Ethnicity, Sex, Smoking History, Socio-economic status
Tumour stage	None
Treatment paradigm	Age, Cancer Type
Time since cancer diagnosis	Age, Ethnicity, Treatment Paradigm
Previous Hospital Visits	Age, Cancer type, Time since cancer diagnosis, treatment paradigm
